# 1486. The Association of Aging-related Comorbidity Burden and Quality of Life Among Women with and without HIV in the U.S.

**DOI:** 10.1093/ofid/ofad500.1322

**Published:** 2023-11-27

**Authors:** Lauren F Collins, Igho Ofotokun, Quincy Greene, Frank J Palella, Susanna Naggie, Elizabeth F Topper, Kathryn Anastos, Audrey L French, Seble Kassaye, Tonya Taylor, Margaret A Fischl, Adaora A Adimora, Mirjam-Colette Kempf, Phyllis C Tien, Anandi N Sheth, Cyra Christina Mehta

**Affiliations:** Emory University School of Medicine, Division of Infectious Diseases, Atlanta, Georgia; Emory University School of Medicine , Lilburn, GA; Emory Rollins School of Public Health, Atlanta, Georgia; Division of Infectious Diseases, Northwestern University, Feinberg School of Medicine, Chicago, Illinois, Chicago, IL; DCRI/ School of Medicine, Durham, North Carolina; Johns Hopkins Bloomberg School of Public Health, Baltimore, Maryland; Department of Medicine, Albert Einstein College of Medicine, Bronx, New York, Bronx, NY; Stroger Hospital of Cook County, Chicago, Illinois; Georgetown University Medical Center, Washington, DC, Washington, DC; SUNY Downstate Medical Center, Brooklyn, NY; Division of Infectious Diseases, University of Miami Miller School of Medicine, Miami, Florida, Miami, FL; Department of Medicine, University of North Carolina at Chapel Hill, Chapel Hill, North Carolina; University of Alabama at Birmingham, Birmingham, Alabama; University of California, San Francisco, San Francisco, California; Emory University School of Medicine, Atlanta, Georgia; Emory University School of Medicine, Atlanta, Georgia

## Abstract

**Background:**

Women with HIV (WWH) are at higher and earlier risk of developing aging-related non-AIDS comorbidities (NACM) compared to those without HIV; however, the impact on health-related quality of life (QoL) is largely unknown.

**Methods:**

We analyzed data from the Women’s Interagency HIV Study to evaluate the effect of comorbidity burden (total NACM of 10 assessed) on nine QoL domains and a summary QoL index (QoLI; measured by the MOS-HIV instrument). We included women followed on/after 2009 (when >80% of WWH reported antiretroviral therapy use) and ascertained covariates, NACM, and QoL through the last study visit by 03/2018. Unadjusted (HIV, age, NACM burden or prevalence separately), partially adjusted (unadjusted + all possible interaction terms) and adjusted (partially adjusted + covariates) linear regression assessed impact on QoL.

**Results:**

Among 3036 women (2173 HIV+, 863 HIV-), median age was 50 (Q1-Q3 43-56) yrs, 66% were Black, 51% had annual income < $12K, and mean (sd) NACM burden was 3.4 (2.2). In unadjusted models, each additional NACM decreased the mean QoLI by -4.4 (95%CI -4.7,-4.1; *p*< 0.0001). Mean QoLI did not differ in WWH vs without HIV (68 vs 69, *p*=0.40) but decreased with older age (< 40, 40-49, 50-59, 60-69 yrs): 75 (95%CI 73,77), 71 (70,73), 65 (63,66), 63 (61,65), respectively (*p*< 0.0001). Unadjusted QoLI was negatively associated with each prevalent NACM (**Table**). NACM burden was associated with all nine QoL domains in unadjusted models: physical function, role function, energy/fatigue, social function, cognitive function, emotional well-being, health perception, pain, and perceived health index. In the partially adjusted model, the impact of NACM burden on QoLI was modified by age (*p*=0.02) but not HIV serostatus (*p*=0.40) (**Figure**). In covariate-adjusted models (race, BMI, cigarette, alcohol or crack use, menopausal status, socioeconomic status), NACM burden was associated with QoLI (*p*< 0.0001) but age and HIV did not modify this effect (age*HIV*NACM burden, *p*=0.83).

Summary quality of life index and aging-related comorbidity burden by HIV serostatus and age

**Figure:**
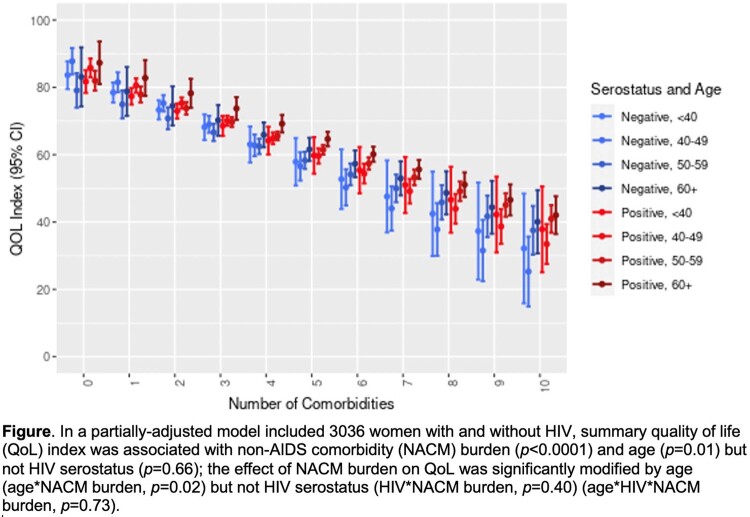

**Figure:**
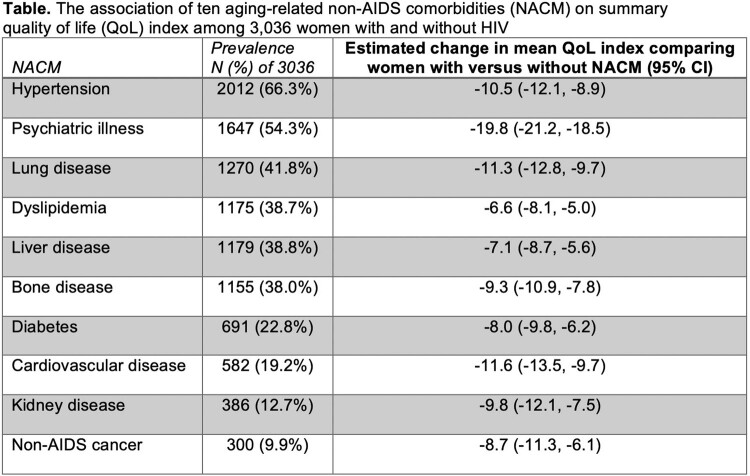

**Conclusion:**

Among women with a high prevalence of multimorbidity, HIV, and health disparities, total comorbidity burden was associated with QoL, independent of age or HIV serostatus. Research is needed to optimize multimorbidity screening and prevention strategies in this population.

**Disclosures:**

**Lauren F. Collins, MD, MSc**, Curio Science: Honoraria **Igho Ofotokun, MD, MSc, FIDSA**, Merck: Grant/Research Support **Phyllis C. Tien, MD, MSc**, Merck: Grant/Research Support **Cyra Christina Mehta, PhD, MSPH**, Merck: Grant/Research Support

